# [(1*S*,2*S*)-2-(1-{[2-(2-Oxidobenzyl­idene­amino)­cyclo­hex­yl]imino}­eth­yl)phenolato-κ^4^ *O*,*N*,*N*′,*O*′]copper(II)

**DOI:** 10.1107/S1600536810022889

**Published:** 2010-06-18

**Authors:** Nura Suleiman Gwaram, Hamid Khaledi, Hapipah Mohd Ali

**Affiliations:** a Department of Chemistry, University of Malaya, 50603 Kuala Lumpur, Malaysia

## Abstract

In the title compound, [Cu(C_21_H_22_N_2_O_2_)], the cyclo­hexyl ring adopts a chair conformation with the two imine groups linked at equatorial positions. The Cu^II^ ion is coordinated by two N atoms and two O atoms from the bis-Schiff base ligand in a slightly distorted square-planar geometry. The dihedral angle between the two benzene rings is 45.89 (9)°. The crystal structure is devoid of any classical hydrogen bonds. However, inter­molecular C—H⋯O inter­actions are present and stabilize the structure.

## Related literature

For the crystal structures of a similar symmetrical compound see: Yao *et al.* (1997 ▶). For metal complexes of unsymmetrical bis-Schiff bases, see: Lashanizadegan & Boghaei (2002 ▶); Rabie *et al.* (2008 ▶).
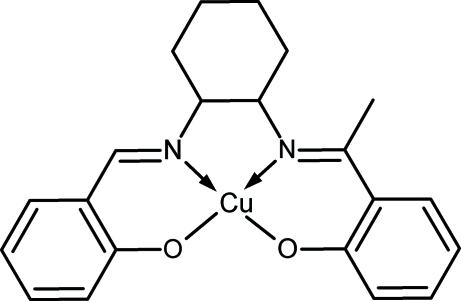


## Experimental

### 

#### Crystal data

[Cu(C_21_H_22_N_2_O_2_)]
*M* *_r_* = 397.95
Monoclinic, 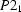

*a* = 9.6699 (3) Å
*b* = 7.7324 (2) Å
*c* = 12.1847 (4) Å
β = 111.649 (2)°
*V* = 846.80 (4) Å^3^
*Z* = 2
Mo *K*α radiation
μ = 1.31 mm^−1^
*T* = 100 K
0.20 × 0.10 × 0.03 mm

#### Data collection

Bruker APEXII CCD diffractometer
Absorption correction: multi-scan (*SADABS*; Sheldrick, 1996 ▶) *T*_min_ = 0.780, *T*_max_ = 0.962
9232 measured reflections
4573 independent reflections
3542 reflections with *I* > 2σ(*I*)
*R*_int_ = 0.053

#### Refinement

*R*[*F* ^2^ > 2σ(*F* ^2^)] = 0.048
*wR*(*F* ^2^) = 0.091
*S* = 0.97
4573 reflections
236 parameters
1 restraint
H-atom parameters constrained
Δρ_max_ = 0.39 e Å^−3^
Δρ_min_ = −0.37 e Å^−3^
Absolute structure: Flack (1983 ▶), 2036 Friedel pairs
Flack parameter: 0.050 (15)

### 

Data collection: *APEX2* (Bruker, 2007 ▶); cell refinement: *SAINT* (Bruker, 2007 ▶); data reduction: *SAINT*; program(s) used to solve structure: *SHELXS97* (Sheldrick, 2008 ▶); program(s) used to refine structure: *SHELXL97* (Sheldrick, 2008 ▶); molecular graphics: *X-SEED* (Barbour, 2001 ▶); software used to prepare material for publication: *publCIF* (Westrip, 2010 ▶).

## Supplementary Material

Crystal structure: contains datablocks I, global. DOI: 10.1107/S1600536810022889/pv2290sup1.cif

Structure factors: contains datablocks I. DOI: 10.1107/S1600536810022889/pv2290Isup2.hkl

Additional supplementary materials:  crystallographic information; 3D view; checkCIF report

## Figures and Tables

**Table 1:** Hydrogen-bond geometry (Å, °)

*D*—H⋯*A*	*D*—H	H⋯*A*	*D*⋯*A*	*D*—H⋯*A*
C9—H9⋯O1^i^	1.00	2.47	3.403 (6)	155
C10—H10*B*⋯O2^ii^	0.99	2.45	3.366 (4)	154

Symmetry codes: (i) 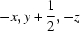; (ii) 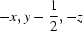.

**Fig. 1.**
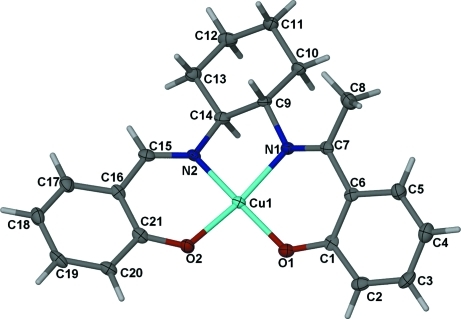
Thermal ellipsoid plot of the title compound at the 50% probability level.

## supplementary crystallographic information

### Comment

The structure of the title complex is shown in Fig. 1. The crystal structures of a similar symmetrical compound (Yao *et al.*, 1997) as well as metal complexes of unsymmetrical bis-schiff bases (Lashanizadegan *et al.,* 2002; Rabie *et al.*, 2008) have been reported.

There are no classical hydrogen bonds observed in this structure. However, there are two C—H···O type inter-molecular interactions, C9–H9..O1 and C10–H10B..O2, observed (Tab. 1) which stabilize the crystal structure.

### Experimental

To an ethanolic solution (10 ml) of 1,2-diaminohexane (0.224 g, 2 mmol) was added a solution of 2-hydroxyacetophenone (0.28 g, 2 mmol) in the same solvent (10 ml). The mixture was stirred at room temperature for 15 minutes, followed by addition of 2-hydroxybenzaldehyde (0.252 g, 2 mmol) in ethanol (10 ml). The resulting yellow solution was stirred for 3 h. Then a solution of copper (II) acetate monohydrate (0.4 g, 2 mmol) in a minimum amount of ethanol was added and the solution was set aside for one day whereupon the green crystals of the title compound were obtained.

### Refinement

Hydrogen atoms were placed at calculated positions (C—H 0.95–1.00 Å), and were treated as riding on their parent atoms with *U*_iso_(H) set to 1.2–1.5*U*_eq_(C). An absolute structure was determined using the Flack (1983) method.

### Crystal data

**Table:**

[Cu(C_21_H_22_N_2_O_2_)]	*F*(000) = 414
*M**_r_* = 397.95	*D*_x_ = 1.561 Mg m^−^^3^
Monoclinic, *P*2_1_	Mo *K*α radiation, λ = 0.71073 Å
Hall symbol: P 2yb	Cell parameters from 1422 reflections
*a* = 9.6699 (3) Å	θ = 3.2–23.5°
*b* = 7.7324 (2) Å	µ = 1.31 mm^−^^1^
*c* = 12.1847 (4) Å	*T* = 100 K
β = 111.649 (2)°	Block, green
*V* = 846.80 (4) Å^3^	0.20 × 0.10 × 0.03 mm
*Z* = 2	

### Data collection

**Table:**

Bruker APEXII CCD diffractometer	4573 independent reflections
Radiation source: fine-focus sealed tube	3542 reflections with *I* > 2σ(*I*)
graphite	*R*_int_ = 0.053
φ and ω scans	θ_max_ = 29.6°, θ_min_ = 1.8°
Absorption correction: multi-scan (*SADABS*; Sheldrick, 1996)	*h* = −13→13
*T*_min_ = 0.780, *T*_max_ = 0.962	*k* = −10→10
9232 measured reflections	*l* = −16→16

### Refinement

**Table:**

Refinement on *F*^2^	Secondary atom site location: difference Fourier map
Least-squares matrix: full	Hydrogen site location: inferred from neighbouring sites
*R*[*F*^2^ > 2σ(*F*^2^)] = 0.048	H-atom parameters constrained
*wR*(*F*^2^) = 0.091	*w* = 1/[σ^2^(*F*_o_^2^) + (0.0281*P*)^2^] where *P* = (*F*_o_^2^ + 2*F*_c_^2^)/3
*S* = 0.97	(Δ/σ)_max_ < 0.001
4573 reflections	Δρ_max_ = 0.39 e Å^−^^3^
236 parameters	Δρ_min_ = −0.37 e Å^−^^3^
1 restraint	Absolute structure: Flack (1983), 2036 Friedel pairs
Primary atom site location: structure-invariant direct methods	Flack parameter: 0.050 (15)

### Special details

**Table:**

Geometry. All e.s.d.'s (except the e.s.d. in the dihedral angle between two l.s. planes) are estimated using the full covariance matrix. The cell e.s.d.'s are taken into account individually in the estimation of e.s.d.'s in distances, angles and torsion angles; correlations between e.s.d.'s in cell parameters are only used when they are defined by crystal symmetry. An approximate (isotropic) treatment of cell e.s.d.'s is used for estimating e.s.d.'s involving l.s. planes.
Refinement. Refinement of *F*^2^ against ALL reflections. The weighted *R*-factor *wR* and goodness of fit *S* are based on *F*^2^, conventional *R*-factors *R* are based on *F*, with *F* set to zero for negative *F*^2^. The threshold expression of *F*^2^ > σ(*F*^2^) is used only for calculating *R*-factors(gt) *etc*. and is not relevant to the choice of reflections for refinement. *R*-factors based on *F*^2^ are statistically about twice as large as those based on *F*, and *R*- factors based on ALL data will be even larger.

### Fractional atomic coordinates and isotropic or equivalent isotropic displacement parameters (Å^2^)

**Table:**

	*x*	*y*	*z*	*U*_iso_*/*U*_eq_	
Cu1	0.09681 (4)	0.25280 (6)	0.05446 (3)	0.01389 (10)	
O1	0.1603 (3)	0.2029 (3)	−0.0696 (2)	0.0199 (7)	
O2	0.2941 (3)	0.3130 (3)	0.1540 (2)	0.0179 (6)	
N1	−0.1155 (3)	0.2570 (8)	−0.0484 (2)	0.0158 (5)	
N2	0.0262 (3)	0.2546 (7)	0.1826 (2)	0.0148 (5)	
C1	0.0826 (4)	0.2231 (4)	−0.1822 (3)	0.0146 (8)	
C2	0.1533 (4)	0.1917 (5)	−0.2626 (4)	0.0210 (9)	
H2	0.2528	0.1501	−0.2330	0.025*	
C3	0.0850 (4)	0.2184 (5)	−0.3815 (3)	0.0239 (10)	
H3	0.1355	0.1914	−0.4331	0.029*	
C4	−0.0588 (4)	0.2855 (5)	−0.4267 (3)	0.0250 (11)	
H4	−0.1056	0.3099	−0.5085	0.030*	
C5	−0.1319 (4)	0.3158 (5)	−0.3509 (3)	0.0206 (8)	
H5	−0.2306	0.3595	−0.3825	0.025*	
C6	−0.0671 (4)	0.2850 (4)	−0.2290 (3)	0.0142 (9)	
C7	−0.1594 (4)	0.3112 (4)	−0.1567 (3)	0.0143 (7)	
C8	−0.3037 (4)	0.4067 (5)	−0.2152 (4)	0.0250 (10)	
H8A	−0.3788	0.3272	−0.2664	0.038*	
H8B	−0.2886	0.5021	−0.2625	0.038*	
H8C	−0.3378	0.4531	−0.1546	0.038*	
C9	−0.2129 (3)	0.2699 (6)	0.0225 (3)	0.0135 (7)	
H9	−0.2298	0.3943	0.0363	0.016*	
C10	−0.3627 (4)	0.1763 (5)	−0.0305 (3)	0.0185 (8)	
H10A	−0.4222	0.2286	−0.1078	0.022*	
H10B	−0.3457	0.0531	−0.0438	0.022*	
C11	−0.4481 (4)	0.1894 (5)	0.0520 (3)	0.0198 (8)	
H11A	−0.4679	0.3125	0.0631	0.024*	
H11B	−0.5450	0.1296	0.0162	0.024*	
C12	−0.3605 (4)	0.1086 (5)	0.1712 (4)	0.0223 (9)	
H12A	−0.3504	−0.0172	0.1611	0.027*	
H12B	−0.4156	0.1250	0.2247	0.027*	
C13	−0.2067 (4)	0.1891 (5)	0.2264 (3)	0.0189 (8)	
H13A	−0.1487	0.1261	0.2998	0.023*	
H13B	−0.2163	0.3110	0.2474	0.023*	
C14	−0.1252 (4)	0.1816 (5)	0.1409 (3)	0.0164 (8)	
H14	−0.1162	0.0566	0.1234	0.020*	
C15	0.0955 (4)	0.3110 (4)	0.2870 (3)	0.0174 (8)	
H15	0.0441	0.3094	0.3401	0.021*	
C16	0.2456 (4)	0.3770 (4)	0.3309 (3)	0.0155 (8)	
C17	0.3008 (4)	0.4505 (5)	0.4447 (3)	0.0206 (9)	
H17	0.2403	0.4492	0.4908	0.025*	
C18	0.4390 (4)	0.5235 (5)	0.4905 (4)	0.0221 (9)	
H18	0.4736	0.5739	0.5670	0.026*	
C19	0.5288 (4)	0.5227 (5)	0.4230 (4)	0.0222 (9)	
H19	0.6250	0.5734	0.4539	0.027*	
C20	0.4793 (4)	0.4495 (5)	0.3123 (3)	0.0187 (8)	
H20	0.5437	0.4474	0.2694	0.022*	
C21	0.3353 (4)	0.3770 (5)	0.2605 (3)	0.0174 (8)	

### Atomic displacement parameters (Å^2^)

**Table:**

	*U*^11^	*U*^22^	*U*^33^	*U*^12^	*U*^13^	*U*^23^
Cu1	0.01095 (17)	0.01506 (18)	0.0154 (2)	0.0002 (3)	0.00449 (14)	0.0004 (3)
O1	0.0160 (13)	0.0234 (17)	0.0211 (15)	0.0011 (10)	0.0077 (12)	−0.0015 (10)
O2	0.0134 (12)	0.0244 (14)	0.0159 (14)	−0.0023 (11)	0.0054 (11)	−0.0004 (10)
N1	0.0127 (13)	0.0195 (13)	0.0160 (13)	0.000 (2)	0.0061 (11)	−0.002 (2)
N2	0.0099 (12)	0.0176 (12)	0.0156 (13)	0.002 (2)	0.0033 (10)	0.000 (2)
C1	0.0217 (17)	0.004 (2)	0.0186 (18)	−0.0023 (14)	0.0084 (15)	−0.0016 (13)
C2	0.0205 (19)	0.0154 (18)	0.030 (2)	0.0004 (15)	0.0132 (18)	−0.0034 (16)
C3	0.033 (2)	0.020 (3)	0.025 (2)	−0.0004 (17)	0.0173 (18)	−0.0067 (16)
C4	0.033 (2)	0.024 (3)	0.0184 (19)	−0.0016 (18)	0.0100 (17)	−0.0004 (16)
C5	0.0200 (19)	0.0220 (18)	0.019 (2)	−0.0013 (16)	0.0060 (16)	0.0010 (15)
C6	0.0173 (17)	0.007 (2)	0.0190 (18)	−0.0004 (14)	0.0078 (14)	−0.0013 (13)
C7	0.0124 (17)	0.0111 (16)	0.0185 (19)	−0.0032 (13)	0.0045 (15)	−0.0039 (14)
C8	0.019 (2)	0.028 (2)	0.024 (2)	−0.0011 (18)	0.0031 (18)	−0.0012 (17)
C9	0.0119 (14)	0.0145 (19)	0.0157 (16)	0.0038 (18)	0.0071 (12)	0.0025 (18)
C10	0.0129 (18)	0.0211 (19)	0.021 (2)	−0.0027 (16)	0.0060 (16)	−0.0062 (16)
C11	0.0145 (18)	0.0249 (19)	0.021 (2)	−0.0025 (15)	0.0072 (17)	−0.0032 (15)
C12	0.017 (2)	0.021 (2)	0.032 (3)	−0.0038 (17)	0.0125 (19)	0.0007 (17)
C13	0.0188 (19)	0.0209 (18)	0.019 (2)	0.0016 (15)	0.0094 (16)	0.0028 (15)
C14	0.0138 (18)	0.0137 (17)	0.022 (2)	0.0013 (15)	0.0071 (16)	0.0000 (15)
C15	0.0136 (18)	0.0157 (18)	0.023 (2)	0.0034 (14)	0.0070 (16)	0.0025 (15)
C16	0.0151 (19)	0.0120 (17)	0.016 (2)	−0.0011 (15)	0.0023 (15)	0.0047 (14)
C17	0.019 (2)	0.0207 (19)	0.018 (2)	0.0039 (16)	0.0023 (17)	0.0016 (16)
C18	0.021 (2)	0.021 (2)	0.019 (2)	−0.0014 (17)	0.0010 (17)	−0.0038 (17)
C19	0.013 (2)	0.017 (2)	0.028 (2)	−0.0020 (16)	−0.0015 (17)	0.0030 (17)
C20	0.0131 (19)	0.0179 (19)	0.024 (2)	0.0001 (15)	0.0049 (17)	0.0045 (16)
C21	0.0160 (19)	0.0141 (18)	0.020 (2)	−0.0014 (15)	0.0049 (16)	0.0050 (15)

### Geometric parameters (Å, °)

**Table:**

Cu1—O1	1.870 (2)	C9—H9	1.0000
Cu1—O2	1.903 (2)	C10—C11	1.522 (5)
Cu1—N2	1.921 (2)	C10—H10A	0.9900
Cu1—N1	1.972 (3)	C10—H10B	0.9900
O1—C1	1.307 (4)	C11—C12	1.519 (5)
O2—C21	1.306 (4)	C11—H11A	0.9900
N1—C7	1.298 (5)	C11—H11B	0.9900
N1—C9	1.498 (4)	C12—C13	1.521 (5)
N2—C15	1.277 (4)	C12—H12A	0.9900
N2—C14	1.474 (4)	C12—H12B	0.9900
C1—C2	1.407 (5)	C13—C14	1.522 (5)
C1—C6	1.429 (5)	C13—H13A	0.9900
C2—C3	1.368 (5)	C13—H13B	0.9900
C2—H2	0.9500	C14—H14	1.0000
C3—C4	1.394 (5)	C15—C16	1.443 (5)
C3—H3	0.9500	C15—H15	0.9500
C4—C5	1.374 (5)	C16—C17	1.409 (5)
C4—H4	0.9500	C16—C21	1.427 (5)
C5—C6	1.403 (5)	C17—C18	1.366 (5)
C5—H5	0.9500	C17—H17	0.9500
C6—C7	1.481 (5)	C18—C19	1.400 (6)
C7—C8	1.506 (5)	C18—H18	0.9500
C8—H8A	0.9800	C19—C20	1.376 (5)
C8—H8B	0.9800	C19—H19	0.9500
C8—H8C	0.9800	C20—C21	1.415 (5)
C9—C10	1.533 (5)	C20—H20	0.9500
C9—C14	1.538 (5)		
			
O1—Cu1—O2	90.86 (11)	C9—C10—H10A	109.6
O1—Cu1—N2	168.46 (17)	C11—C10—H10B	109.6
O2—Cu1—N2	93.06 (11)	C9—C10—H10B	109.6
O1—Cu1—N1	93.73 (11)	H10A—C10—H10B	108.1
O2—Cu1—N1	164.81 (18)	C12—C11—C10	111.0 (3)
N2—Cu1—N1	85.27 (10)	C12—C11—H11A	109.4
C1—O1—Cu1	126.2 (2)	C10—C11—H11A	109.4
C21—O2—Cu1	126.4 (2)	C12—C11—H11B	109.4
C7—N1—C9	121.7 (3)	C10—C11—H11B	109.4
C7—N1—Cu1	121.6 (2)	H11A—C11—H11B	108.0
C9—N1—Cu1	111.31 (19)	C11—C12—C13	111.4 (3)
C15—N2—C14	124.3 (3)	C11—C12—H12A	109.4
C15—N2—Cu1	126.9 (3)	C13—C12—H12A	109.4
C14—N2—Cu1	108.9 (2)	C11—C12—H12B	109.4
O1—C1—C2	118.1 (3)	C13—C12—H12B	109.4
O1—C1—C6	124.3 (3)	H12A—C12—H12B	108.0
C2—C1—C6	117.4 (3)	C12—C13—C14	110.5 (3)
C3—C2—C1	122.9 (4)	C12—C13—H13A	109.6
C3—C2—H2	118.6	C14—C13—H13A	109.6
C1—C2—H2	118.6	C12—C13—H13B	109.6
C2—C3—C4	119.7 (3)	C14—C13—H13B	109.6
C2—C3—H3	120.1	H13A—C13—H13B	108.1
C4—C3—H3	120.1	N2—C14—C13	116.7 (3)
C5—C4—C3	118.9 (4)	N2—C14—C9	106.7 (3)
C5—C4—H4	120.5	C13—C14—C9	112.3 (3)
C3—C4—H4	120.5	N2—C14—H14	106.9
C4—C5—C6	122.9 (4)	C13—C14—H14	106.9
C4—C5—H5	118.5	C9—C14—H14	106.9
C6—C5—H5	118.5	N2—C15—C16	125.2 (3)
C5—C6—C1	118.0 (3)	N2—C15—H15	117.4
C5—C6—C7	118.4 (3)	C16—C15—H15	117.4
C1—C6—C7	123.5 (3)	C17—C16—C21	119.9 (3)
N1—C7—C6	121.2 (3)	C17—C16—C15	118.1 (4)
N1—C7—C8	122.5 (3)	C21—C16—C15	122.0 (3)
C6—C7—C8	116.3 (3)	C18—C17—C16	121.9 (4)
C7—C8—H8A	109.5	C18—C17—H17	119.1
C7—C8—H8B	109.5	C16—C17—H17	119.1
H8A—C8—H8B	109.5	C17—C18—C19	118.9 (4)
C7—C8—H8C	109.5	C17—C18—H18	120.6
H8A—C8—H8C	109.5	C19—C18—H18	120.6
H8B—C8—H8C	109.5	C20—C19—C18	120.7 (4)
N1—C9—C10	115.0 (3)	C20—C19—H19	119.6
N1—C9—C14	105.4 (3)	C18—C19—H19	119.6
C10—C9—C14	107.1 (3)	C19—C20—C21	122.0 (4)
N1—C9—H9	109.7	C19—C20—H20	119.0
C10—C9—H9	109.7	C21—C20—H20	119.0
C14—C9—H9	109.7	O2—C21—C20	118.9 (4)
C11—C10—C9	110.4 (3)	O2—C21—C16	124.5 (3)
C11—C10—H10A	109.6	C20—C21—C16	116.6 (3)

### Hydrogen-bond geometry (Å, °)

**Table:**

*D*—H···*A*	*D*—H	H···*A*	*D*···*A*	*D*—H···*A*
C9—H9···O1^i^	1.00	2.47	3.403 (6)	155
C10—H10B···O2^ii^	0.99	2.45	3.366 (4)	154

Symmetry codes: (i) −*x*, *y*+1/2, −*z*; (ii) −*x*, *y*−1/2, −*z*.
